# Integrated science, technology, engineering, and mathematics project-based learning for physics learning from neuroscience perspectives

**DOI:** 10.3389/fpsyg.2023.1136246

**Published:** 2023-06-19

**Authors:** Lorna Uden, Fauziah Sulaiman, Gregory S. Ching, Jeffry Juan Rosales

**Affiliations:** ^1^School of Computing, Faculty of Computing and Digital Technologies, Staffordshire University, Stoke-on-Trent, United Kingdom; ^2^Faculty of Science and Natural Resources, Universiti Malaysia Sabah, Kota Kinabalu, Sabah, Malaysia; ^3^Graduate Institute of Educational Administration and Policy, National ChengChi University, New Taipei, Taiwan

**Keywords:** integrated STEM-PjBL, students’ beliefs, physics, learning physics, educational neuroscience, framework

## Abstract

For many students, learning physics is difficult because of its abstractness. To help students to learn physics, we have developed the Integrated Science, Technology, Engineering, and Mathematics Projects Based Learning (STEM-PjBL) method based on principles from neuroscience. We believe that incorporating principles from educational neuroscience would help students learn better. This paper describes our experiments of implementing the integrated STEM-PjBL Module in physics, i.e., classical mechanics, to secondary school students in Malaysia and South Korea. The study consists of two groups of students: the experiment group, 77 in total, comprising those who have undergone the integrated STEM-PjBL, and the control group, again 77 in total, who experienced the traditional approach. The Colorado Learning Attitudes Science Survey (CLASS) was conducted for the two groups on students’ beliefs about physics and learning physics before and after the implementation. The paired sample *t*-test from the pre-survey and post-survey shows that the integrated STEM-PjBL group has a more positive shift in belief about physics and learning physics than the traditional group. The results of the independent samples *t*-test for students’ beliefs about physics and learning physics, compared with the post-survey between the experimental group and the traditional group for both Malaysian and Korean perspectives, show that the experimental group has a higher mean compared to the traditional group. This paper explains why the integrated STEM-PjBL has improved students’ beliefs about physics and learning physics, from the neuroscience education perspective. Finally, the paper concludes with guidelines for teachers who wish to implement the integrated STEM-PjBL in the classroom.

## 1. Introduction

Physics is a complex subject to learn (Veronika et al., 2017). Students often have this perception, and they also have low confidence in learning physics, resulting in fewer students taking up physics at school (Fatin et al., 2012). According to Dolin’s study (as cited in Angell et al., 2004), learning physics requires students to learn many types of representation, such as experiments, graphs, and mathematical symbols. Students must understand and learn the transformation between all these representations. Another factor that hinders students from studying Physics is that they are not interested in the subject and feel bored (Hirschfeld, 2012). As a result, most students only managed to obtain an average grade in physics (MoE, 2013–2025; Halim et al., 2018). Factors like lack of teachers’ engagement, lack of class activities that promote learning, teachers’ overload of work that only focuses on finishing the syllabus within the time frame given, and teachers who are not self-confident in teaching practical physics work are the reasons why students stay away from physics. Besides, students’ poor attitudes and no interest toward physics are also factors that contribute to this issue (Josiah, 2013). Most students think physics is boring, difficult, and irrelevant to daily life (Williams et al., 2003). Lack of laboratory facilities and less exposure to practical instruction led to poor achievement of physics in school (Daramola, as cited in Musasia et al., 2012). Teachers also lack exposure to science process skills to carry out activities in class (Rose et al., 2013). Although many realize the importance of physics in school, the teaching and learning of physics is still a great concern in education.

Most students who learn physics for the first-time result in negative shifts in beliefs about physics and learning physics (Madsen et al., 2015). Students with negative beliefs would consider physics to be difficult (Sahin, 2010) and beyond their capabilities to comprehend (Kovanen, 2011). The difficulty in learning physics results in declining enrolment in physics by students in the secondary school (Wang et al., 2017; Sheldrake et al., 2019). Physics instruction is a crucial factor that affects the shift in students’ beliefs about physics and learning physics (Hammer, 1994; Wieman and Perkins, 2005; Madsen et al., 2015). Students who had negative experience are associated with unengaging instruction (Wang et al., 2017). Research has shown that traditional instruction resulted in a negative experience for students when learning physics (Donley and Ashcraft, 1992; Sahin, 2010; Madsen et al., 2015; Hairan et al., 2018). Beliefs about physics and learning physics significantly impact how students’ approach and learn physics (Hammer, 1994; Chang, 2005; Mistades, 2007), and these attitudes are crucial when students first encountered physics. Students who hold positive beliefs about physics and learning physics tend to believe that physics knowledge is a coherent and logical method to understand the world (Madsen et al., 2015). Therefore, identify students’ belief in physics is crucial before mentioning their interests, attitudes, engagement, and motivation.

Research-based instruction with an explicit focus on inquiry, modeling building instruction, experimentation and real-world contexts result in a positive experience for students in physics and learning physics (Madsen et al., 2015). It is our belief that integrated STEM-PjBL physics teaching could be used to improve students’ beliefs about physics and learning physics. Research has been done regarding the acceptance of learning physics, e.g., students’ interest decreased in learning physics at secondary school (O’Neill and Mcloughlin, 2021), students’ preferences for learning physics at the college level declined (Riskawati and Marisda, 2022); students’ beliefs toward learning physics and its influencing factors, i.e., students’ beliefs to learn physics, students attitudes toward physics, and influence of cultural belief on students to learn physics (Chala et al., 2020). Researchers suggested that teachers should change their way of teaching physics and learning style to boost students’ interest at the secondary level (Ziad et al., 2021). However, as far as we know, there has been no research carried out to discuss the shift in belief about physics and learning physics, particularly from the neuroscience perspective.

The aim of this study was to investigate the effectiveness of integrated STEM-PjBL physics method to help students to improve their beliefs about physics and learning physics among Malaysian and Korean students. The objectives of the study are:

1. To investigate the effectiveness of integrated STEM-PjBL physics method to improve students’ beliefs about physics and learning physics.
2. To compare beliefs about physics and learning physics between Malaysian students and Korean students after the implementation of integrated STEM-PjBL physics module.
3. To discuss the findings from the principles of educational neuroscience.

Educators and schools around the world are increasingly using the knowledge, techniques, and programs developed from a new understanding of how our brains learn; that is neuroscience in their classrooms. Educational neurosciences empower teachers with a new understanding about how students learn. Principles from educational neuroscience have important implications to understanding learning. In our research we have incorporated the principles of neuroscience in our STEM-PjBL to teach physics and explain why it was successful. Based on the research findings from our study, guidelines based on educational neuroscience will be provided to guide teachers how to design effective STEM-PjBL.

This paper begins with a brief review of teaching and learning and why we proposed STEM-PjBL. A brief overview of Project Based Learning for STEM and neuroscience and their implications for teaching and learning are given. This is followed by description of the case study and methodology. Subsequent sections present the results. This is followed by discussion and guidelines to design STEM-PjBL based on principles from neuroscience. The paper concludes with the conclusion and recommendations for further studies.

## 2. Literature review

Physics is well-known as a driving force for innovation and the development of new technologies (Lee and Kim, 2018). This is because physics has a strong connection to the integrated STEM elements (Bunyamin et al., 2020). To ensure students have a good understanding of physics, they must have a strong foundation in understanding classical mechanics concepts, which are taught starting in secondary education (Hairan et al., 2018). Students who understand classical mechanics concepts are known to have positive beliefs about physics and learning (Kiong and Sulaiman, 2010; Sahin, 2010; Madsen et al., 2015). Applying appealing physics instruction to students can help students to understand classical mechanics concepts better (Aviyanti, 2020), experience a positive shift in beliefs about physics and learning physics as well as having a personal interest, sense making and effort, real world connections, conceptual connections, applied conceptual understanding, problem solving in general, problem solving confidence and problem solving sophistication (Adams et al., 2006) and resulting in having a desire to pursue STEM majors and careers (Wang et al., 2017).

### 2.1. Ways to teach physics

The ways of teaching physics have been evolving for almost 200 years. There are many approaches educators, teachers, and lecturers use to teach physics across levels, e.g., through experiments and collaborative learning in physics (Reiner, 1998), through a contextual approach (Wilkinson, 1999), and real-life context for learning physics (1999). Entering the millennial, more approaches were introduced, including; problem-based learning through online (Atan et al., 2005), active learning strategy (Karamustafaoglu, 2009), teaching physics using PhET simulations (Wieman, 2010), using analogies and examples to overcome misconceptions in physics (Brown, 2014), individual and group learning in physics (Bocaneala, 2015), project-based learning to teach pre-service teachers (Olzan and Bevins, 2016), teaching physics trough practical work (Lee and Fauziah, 2018), teaching physics using history (Karam and Lima, 2022); use of anecdotes to show how physics works (Parmar, 2022) and many more. To promote the interest of students learning, a new approach is needed to meet with the demand of today’s employers’ needs.

### 2.2. A new approach to learn physics

Employers nowadays are demanding thinking, communication, team, and problem-solving skills. Few of these skills are evident in classroom teaching, with students memorizing facts for regurgitation. Traditional teaching is typically characterized by students sitting passively in the classroom as receivers of information, and the teacher is the sole information giver. There is no interaction between students and teachers. Teaching is typically textbook-driven, and information is often presented as discrete parts. The role of the teacher is to transmit information to the passive students. This approach creates many problems. Firstly, students regurgitate what they have learned without understanding. Secondly, students often perceive what they have learned as detached from the real world (Uden and Beaumont, 2006). Thirdly, there is no interaction between the teacher and other students. Fourthly, students rely on the teacher to tell them what to think and learn. Fifthly, students merely learn content without problem-solving skills.

To meet the demand of employers for graduates possessing the problem-solving, communication, critical thinking, team working and self-directed learning skills, there is an urgent need to change the way we teach. This is particularly important for the teaching of physics to students. Physics is a very abstract subject. Students find it hard to learn because of its abstractness. Project-based learning is an alternative approach to teaching and learning that would enable students to acquire the skills they needed in life and those demanded by employers.

### 2.3. The integrated STEM-PjBL

There are several studies in the literature reporting different aspects of project-based learning (PjBL) pedagogy, for instance, PjBL for in-service teachers development to provide effective teachers instruction (Holubova, 2008); PjBL to analyze student cognitive achievement in learning physics (Santyasa et al., 2020); examine the impact of PjBL games on students’ physics achievement in physics (Baran et al., 2018); Integrating PjBL with E-Learning through lesson study activities to improved student quality of learning (Widyaningsih and Yusuf, 2020) and PjBL on self-efficacy among high-school physics students (Samsudin et al., 2017). However, the effect of STEM-PjBL implementation on students’ belief in physics and learning physics at the high school level still needs proof.

PjBL is an instructional methodology based on the constructivist learning theory, in which students learn important skills by doing actual projects (Holubova, 2008). Solving authentic problems in real-world situations is a crucial activity where students apply core academic skills and creativity. Final products such as videos, artwork, reports, photography, music, model construction, live performances, action plans, digital stories, and websites are examples of PjBL artifacts. Normally, they executed the projects using a wide range of tools. On the other hand, STEM education is based on educating students in four specific disciplines, i.e., science, technology, engineering and mathematics into a cohesive learning paradigm based on real-world applications (Sumintono, 2015). Many countries accept STEM education because it provides opportunities to equip students with the knowledge and skills needed in the 21st century and to cope with the challenges of the fourth industrial revolution (Naudé, 2017; Suraya et al., 2017; Brown-Martin, 2018; Türk et al., 2018). For example, Malaysia adopted STEM education by introducing the Malaysian Education Blueprint (2013–2015) in 2013 that aims to raise the existing standard of science and technology education (Bakar et al., 2019). The blueprint introduction is the continuous effort to empower Malaysia to become a developed nation with a STEM-literate society, achieve a targeted highly skilled, qualified STEM workforce and meet the demands of a STEM-driven economy (Shahali et al., 2017). In Korea, the Science, Technology, Engineering, Art and Mathematics (STEAM) STEAM education policy was issued nationwide in 2011 by the Ministry of Education in Korea purposely to promote STEAM education in primary and secondary schools (Kang, 2019). The main goal of STEAM education in Korea is to produce students with the ability to create new ideas or products formed by STEAM competencies purposely to generate a quality STEM workforce, highly technological literate citizens and competent individuals to vitalize the national economy (Jho et al., 2016). STEAM education in Korea is in line with STEM education policy in other countries but with the inclusion of art as another discipline (Kang, 2019).

### 2.4. Neuroscience

Broadly speaking, the concept of neuroscience involves the scientific study of the human brain and the nervous system from a multidisciplinary perspective to determine how it works. Neuroscience is also often referred to as the study of the biological basis for behavior (Squire et al., 2013; Goswami, 2020). Started in the late 20th century as an emerging discipline and constantly evolving, neuroscience is now a multidisciplinary science that integrates many different fields, including psychology, biology, medicine, and many more (Goswami, 2004; Brown, 2019; Sussman, 2021). Neuroscience can be separated into five major branches (Romero, 2019; Meilleur, 2022), such as: systems, medical or clinical, cellular and molecular, cognitive, behavioral, and computational neuroscience.

Essentially, *system neuroscience* is the study of how the human nervous system and the brain relate to each other in terms of how information is encoded or decoded. These processes lead to a wide range of behaviors, including sensory perception, motor control, memory, attention, and language. This field is closely related to *medical* or *clinical neuroscience*, which besides studying the normal functioning of the human nervous system, also examines the various diseases associated with it. Some of the more common disorders include trauma, dementia, Parkinson’s disease, mental illnesses, and a variety of others. Ultimately, medical neuroscience is concerned with treating and preventing these conditions.

*Cellular or molecular neuroscience* involves the study of the human brain’s core cells and neurons. Additionally, it may include the exploration of genes, proteins, and other molecules related to the functioning of the human brain. It is based on these components that studies of brain chemistry are conducted, which are responsible for explaining the processes of perception, learning, and memory. For *cognitive and behavioral neuroscience*, this encompasses our thoughts, behaviors, emotions, and self-awareness. In general, cognitive and behavioral neuroscience focus on how the human brain affects behavior, which can range from psychology to psychiatry. Lastly, *computational neuroscience* involves the use of mathematical, physics and computer science techniques to analyze biological and clinical data on the nervous system. Typically, computational neuroscience involves the use of computers in order to simulate how the human brain functions; more specifically, how information is processed.

Educational neuroscience is an inter-disciplinary and relatively new subject often associated with the science of learning. The goal of educational neuroscience is to improve educational practice by applying findings from brain research into the classrooms. Educational Neuroscience is also referred to as ‘mind, brain and education’ and as ‘neuroeducation.’

Educational neuroscience is helping us to shed light on subjects such as why certain types of learning are more rewarding than others; the plasticity of the brain and what happens when we learn new skills at different ages; ways of enhancing our ability to learn, and the role of digital technologies in learning, along with many others. It has potential impacts to improve educational outcomes by changing factors that influences learning, factors such as motivation, attention, ability to learn, memory, prior knowledge, stress, health and nutrition (Scando review 2022).

A report by the Royal Society in 2011 stated that while education is about enhancing learning, neuroscience is about understanding the mental processes involved in learning. This suggests that which educational practice can be transformed by science, just as medical practice was transformed by science about a century ago.” –.

According to Wikipedia **“**Educational neuroscience also called Mind Brain and Education or Neuroeducation is an emerging scientific field that brings together researchers in cognitive neuroscience, developmental cognitive neuroscience, educational psychology, educational technology, education theory and other related disciplines to explore the interactions between biological processes and education. Researchers in educational neuroscience investigate the neural mechanisms of reading, numerical cognition, attention and their attendant difficulties including dyslexia, dyscalculia and ADHD as they relate to education. Educational neuroscience has received support from both cognitive neuroscientists and educators.

Research in educational neuroscience also link basic findings in cognitive neuroscience with educational technology to help in curriculum implementation for mathematics education and reading education. The aim of educational neuroscience is to generate basic and apply research that will provide a new trans-disciplinary account of learning and teaching, which is capable of informing education. A major goal of educational neuroscience is to bridge the gap between the two fields through a direct dialog between researchers and educators, avoiding the “middlemen of the brain-based learning industry.”

Petitto and Dunbar (2004) argued that educational neuroscience “provides the most relevant level of analysis for resolving today’s core problems in education.” A survey conducted by Howard-Jones et al. (2007) found that teachers and educators were generally enthusiastic about the use of neuroscientific findings in the field of education, and that they felt these findings would be more likely to influence their teaching methodology than curriculum content. A direct link from neuroscience to education is a bridge too far, argued by some researchers (Bruer, 1997; Mason, 2009). They argued that a bridging discipline, such as cognitive psychology or educational psychology provide a better neuroscientific basis for educational practice.

However, many researchers disagreed and argued that the link between education and neuroscience has yet to realize its full potential, and whether through a third research discipline, or through the development of new neuroscience research paradigms and projects, the time is right to apply neuroscientific research findings to education in a practical and meaningful way (Goswami, 2006; Meltzoff et al., 2009).

There are many academic institutions that are beginning to establish research centers focused on educational neuroscience research around the world. One of these is the Center for Educational Neuroscience in London, United Kingdom which is an inter-institutional project between University College, London, Birkbeck and the UCL Institute of Education. The center brings together researchers with expertise in the fields of emotional, conceptual, attentional, language and mathematical development, as well as specialists in education and learning research with the aim of building a new scientific discipline, i.e., Educational Neuroscience in order to ultimately promote better learning” (Wikipedia).

In response to Bowers (2016) criticism of the practical and principled problems with how educational neuroscience may contribute to education, including lack of direct influences on teaching in the classroom. The authors of this paper concur with Gabrieli (2016) that some of his arguments are convincing especially the critique of unsubstantiated claims about the impact of educational neuroscience and the reminder that the primary outcomes of education are behavioral, such as skill in reading or mathematics. There are three major issues. Firstly, educational neuroscience is a basic science that has made unique contributions to basic education research; it is not part of applied classroom instruction. Secondly, educational neuroscience contributes to ideas about education practices that are important for helping vulnerable students. Thirdly, educational neuroscience studies using neuro-imaging have not only revealed for the first time the brain basis of neurodevelopmental differences that have profound influences on educational outcomes but have also identified individual brain differences that predict which students learn more or learn less from various curricula (Gabrieli, 2016). It is our belief that educational neuroscience can inform our understanding of learning, which in turn, choices in educational practice and the design of educational contexts, which can themselves help test and inform the theories from cognitive neuroscience and psychology. Even though educational neuroscience does not support a direct link from neural measurement to classroom practice (Howard-Jones et al., 2016).

#### 2.4.1. Core concepts of neuroscience and educational neuroscience

A major component of neurosciences is explaining how the human brain and nervous system work. From understanding the relationship between brain and behavior to the concepts of learning and memory (Webster, 1999; Bear et al., 2015; Kandel et al., 2021). According to the Society for Neuroscience (2022), it is essential to understand how the brain works and how it is formed, and how it can help guide us through the various changes in our lives. In accordance with the Next Generation Science Standards, neuroscience core concepts (including the basic principles of neuroscience) are being integrated into the various K-12 course subjects. The eight core concepts are as follows (Society for Neuroscience, 2022): the brain is the body’s most complex organ, neurons communicate using both electrical and chemical signals, genetically determined circuits are the foundation of the nervous system, life experiences change the nervous system, intelligence arises as the brain reasons, plans, and solves problems, the brain makes it possible to communicate knowledge through language, the human brain endows us with a natural curiosity to understand how the world works, and fundamental discoveries promote healthy living and treatment of disease. Using these eight core concepts throughout the K-12 curriculum will allow students to gain and learn the most important insights from decades of neuroscience research.

In higher education, the use of computer simulations (or model building) is an effective method in learning and teaching neuroscience (Rabinovich et al., 2006). Through direct engagement within the computer simulations, students are able to receive immediate feedback and reinforcement for their efforts (Av-Ron et al., 2006). Taking advantage of the core concepts, neuroscience, as previously noted, emerges as a multidisciplinary science that integrates many different fields of study that vary in depth and complexity. Therefore, in order to understand human behavior, including its complex functions like thinking and feeling, we must understand how the brain mediates these functions. Importantly, it is pertinent to note that modern neuroscience is multidisciplinary in nature, allowing it to be integrated with a variety of life science disciplines (such as genetics, molecular biology, biochemistry, biophysics and psychology), increasing our understanding of nervous system function and how neuroscience overlaps with other areas of study related to it (such as cognitive science, information science, linguistics, and experimental and clinical psychology).

As for educational neuroscience, which combines the mind, brain, and education with biology, cognitive science, development, and education (Fischer et al., 2010). Feiler and Stabio (2018) identified three emerging themes that are representative of the literature of the past three decades, namely: application of neuroscience to classroom learning, interdisciplinary collaboration, and a translator of languages (pp. 18–20). These themes clearly noted the importance of neuroscience in education (Howard-Jones et al., 2016), dispelling the myth that teachers and students are unable to integrate neuroscience into their teaching (Clement and Lovat, 2012; Bowers, 2016). Quoting the journal *Trends in Neuroscience and Education*: “*Neuroscience is to education what biology is to medicine and physics is to architecture*.” In other words, this does not mean that educational psychology will be replaced by educational neuroscience. In fact, it is very important that educational neuroscience builds on the previous achievements of other disciplines and helps students develop a better understanding of how they learn.

Neuroscience can help teachers to teach in several ways, according to Barnes (2019), these include:

- Improve reading
- Deliver individualized learning for every student
- Help teachers move closer to creating learning environments, rather than simply delivering curriculum content
- Build the learning capacity of each student, so they learn more easily
- Free teachers’ time to teach and add higher value learning opportunities
- Empower teachers with a new understanding about how students learn
- Help students with a range of learning difficulties

Since neuroscience offers many benefits to the learning of physics, it is our belief that by incorporating principles from neuroscience to STEM–PjBL serves as a breaking point to learn classical mechanics with the hope they can improve their beliefs about physics and learning physics STEM knowledge and skills needed in the 21^st^ century.

## 3. Methodology

The quasi-experimental research design was used to collect quantitative data. This research used the two group pre-survey-post-survey of the quasi-experimental research design. The population in this study were Malaysian Form 4 students who learn physics in the secondary school and Korean second-year high school students who learn physics (Book 1). The process of extracting the samples from the population were based on the purposive sampling techniques. The Malaysian sample was selected from four intact groups at two secondary schools in Sabah, Malaysia and the Korean sample was selected from four intact groups at two high schools in Seoul, South Korea. The samples consisted of 88 Malaysian students (i.e., experimental group = 44, control group = 44) and 66 Korean students (i.e., experimental group = 33, control group = 33). The samples were considered homogenous because the participants never experienced learning physics through the integrated STEM-PjBL physics module and the chosen topics in the module were learnt for the first-time during Form 4 and second-year high school, respectively, for both samples.

### 3.1. Research design

This study applied a two-group pre-survey-post-survey design was employed in the quasi-experimental research design which identified as the experimental group and the control group to collect the quantitative data [55]. Both groups were given a pre-survey to measure the dependent variable by using the same instrument a week before the intervention. Then, the experimental group had received the intervention, but the control group did not receive any intervention for 8 weeks of duration. A week after the intervention, both groups were given a post-survey to measure the dependent variable again by using the same instrument. The results of pre-survey and post-survey were examined to identify the improvement of the dependent variable. The framework of the two-group pre-survey-post-survey of the quasi-experimental research design suggested by Harris et al. (2004) used as a reference for this study shown in Table 1.

**Table 1:** Two-group pre-survey-post-survey design.

Group	Implementation		
Experimental	O1_a_	X	O2_a_
Control	O1_b_		O2_b_

*O1_a_ and O1_b_ = Pre-Survey; *X* = Intervention; O2_a_ and O2_b_ = Post-Survey.

### 3.2. The integrated STEM-PjBL physics module

The Integrated STEM-PjBL Physics Module was structured and established following a thorough process by using ADDIE instructional design model. In the Integrated STEM-PjBL Physics Module, some activities may promote students’ personal interest; sense-making and effort; real-world connection, conceptual connections, applied conceptual understanding, problem-solving general, problem-solving confidence, and problem-solving sophistication. These activities need students’ involvement for 8 weeks, e.g., only for the experimental group. First, in groups (3–4 students), students will be given a scenario; then, they must come up with solutions to overcome the learning issue. The Integrated STEM-PjBL Physics Module consists of two chapters, i.e., the Egg Drop Project and the Spaghetti Bridge Project. Both modules will be given to the experiment groups of Form 4 students (Malaysia) and Second-year students (Korea), respectively.

The content of Integrated STEM-PjBL Physics Module was designed based on the PjBL model developed by The Buck Institute of Education (Larmer and Mergendoller, 2010). The PjBL model was used to guide the steps in implementing STEM–PjBL activities and the learning objectives were integrated into the PjBL model. Based on the PjBL model, students had to follow nine (9) steps to achieve the learning objectives for each of STEM-PjBL activity in four (4) weeks of duration. Each step had its own learning activity and students had to accomplish one step before moving to the subsequent step. After completing the first STEM-PjBL activity, students repeated the nine (9) steps of PBL model once again to implement the second STEM-PjBL activity for another four (4) weeks of intervention. The nine (9) steps in implementing STEM-PjBL activities provide guidelines for students to develop the science process. These steps and its connection with both projects, i.e., egg-drop project and spaghetti bridge is shown in Table 2.

**Table 2:** The nine steps and it’s its connection with both projects, i.e., egg-drop project and spaghetti bridge.

Steps	Egg drop project activities	Spaghetti bridge activities
Step 1–build the culture.	Facilitator presents about:	Facilitator presents about:
	STEM-PjBL as an approach to learn physics	STEM-PjBL as an approach to learn physics
	The procedures on how to use the STEM-PjBL physics module	The procedures on how to use the STEM-PjBL physics module
Step 2–group setting–students developed observation skill by planning events in implementing STEM-PjBL activities chronologically after receiving details about the activities.	i. Group formation	i. Group formation
	ii. Establish group rules	ii. Establish group rules
	iii. Define roles of each member	iii. Define roles of each member
Step 3–essential question–students developed communication skill by brainstorming and communicating on draft solutions about the essential question and presented the draft solutions through sketches. Besides that, students developed classification skills by choosing the best design to be developed as a final product by considering the manipulative, responding and constant variables.	How to protect an egg from breaking when it falls from a certain height by using permissible materials; toothpicks, glues and a raw egg?	How to construct a stronger spaghetti bridge that is capable of holding more loads by using permissible materials; spaghetti sticks and glues?
	Based on the essential question, each group:	Based on the essential question, each group:
	i. Brainstorm on the draft solutions	i. Brainstorm on the draft solutions
	ii. Present the ideas through sketches	ii. Present the ideas through sketches
	iii. Choose the best design of the egg protector by comparing variables	iii. Choose the best design of the spaghetti bridge by comparing variables
	iv. Group reflection	iv. Group reflection
Step 4–sustained inquiry–students developed valuing skill by finding additional information about related physics concepts and relating the concepts into their design. The students also developed experimentation skill by constructing prototype and carried out a simple experiment to test the prototype. Students also developed interpretation skill by interpreting the results from the experiment and consequently drawing conclusions to improve the design.	Each group:	Each group:
	i. Find resources and additional information about related physics concept with the egg drop project	i. Find resources and additional information about related physics concept with the spaghetti bridge project
	ii. Construct the prototype	ii. Construct the prototype
	iii. Make improvement by experimenting	iii. Make improvement by experimenting
Step 5–decision making–students developed prediction skill by securing the ultimate design to be developed as final product after discussion was made in the group.	Each group:	Each group:
	i. Compare and reason the results after testing the prototype of the egg protector	i. Compare and reason the results after testing the prototype of the spaghetti bridge
	ii. Discuss and secure the ultimate design to be developed as the final egg protector	ii. Discuss and secure the ultimate design to be developed as the final spaghetti bridge
Step 6–Execute the Solution–students developed communication skill by constructing the final product as planned.	Each group:	Each group:
	i. Construct the final product by using provided materials:–Toothpicks, superglues or hot glue gun and a raw egg	i. Construct the final product by using provided materials:–Spaghetti sticks, superglues or hot glue gun
	ii. Communicate their progress	ii. Communicate their progress
	iii. Group reflection	iii. Group reflection
Step 7–public product–students developed measuring skill by measuring physical quantities by using appropriate instruments and avoid errors when taking measurements. Besides that, students developed experimentation skill by carrying out a simple experiment to test the final product. Students also developed interpretation skill by drawing conclusions based on the results from the experiment.	Each group:	Each group:
	i. Take measurements for the mass of the egg protector, height of the egg protector before dropping and the time traveled for the egg protector before touch the floor without errors.	i. Take measurements for the mass of the spaghetti bridge
	ii. Egg drop testing and public viewing	ii. Spaghetti bridge testing and public viewing
	iii. Interpret the results after the egg drop testing	iii. Interpret the results after the spaghetti bridge testing
	iv. Group reflection	iv. Group reflection
Step 8–assess student learning–students developed forming questions and hypotheses skills by solving physics problems in the module.	Each group:	Each group:
	i. Make connections between the equations of linear motions with the egg drop testing activity to solve physics problems	i. Identify the maximum loads which the spaghetti bridge can hold before the collapse.
	ii. Interpret the motion of the egg protector in the velocity-time graph	ii. Calculate the spaghetti bridge performance
	iii. Make connections between the momentum with the egg drop project	iii. Learn from observation
	iv. Make connections between the impulsive force with the egg drop project	iv. Name the type of bridge constructed in the spaghetti bridge project
	v. Relate the impulsive force with daily life situations:–Safety features in vehicles The use of mattress in high jump	v. Make connections between the effects of a force with the spaghetti bridge project
	vi. Make connections between the kinetic energy with the egg drop project	vi. Make connections between the gravity with the spaghetti bridge project
	vii. Make connections between the gravitational energy with the egg drop project	vii. Make connections between the forces in equilibrium with the spaghetti bridge project
	viii. Make connections between the kinetic energy and the gravitational energy	viii. Relate the gravity and the forces in equilibrium with daily life situations
	ix. Communicate their progress	ix. Communicate their progress
	x. Group reflection	x. Group reflection
Step 9–Evaluate the Experience–students developed communication skill by sharing their opinions, beliefs and attitudes about the STEM-PjBL activities	i. Focus group discussion	i. Focus group discussion
	Share their opinions, beliefs and attitudes about the egg drop project with the other groups	Share their opinions, beliefs and attitudes about the spaghetti bridge project with the other groups
	ii. Group video presentation	ii. Group video presentation
	iii. Group reflection	iii. Group reflection

### 3.3. Data collection procedures

Data was collected quantitatively using The Colorado Learning Attitude about Science Survey (CLASS). CLASS was developed based on the Maryland Physics Expectation Survey (MPEX) (Redish et al., 1998) and the Views about Science Survey (VASS) (Halloun and Hestenes, 1996). It was developed to probe students’ beliefs about physics and learning physics (Adams et al., 2006). CLASS focuses on the aspects of epistemology and student thinking, making it suitable to explore students’ beliefs about the nature of physics knowledge and learning. In addition, CLASS is not course-specific and ideal for students at any level of physics (Perkins et al., 2006). CLASS consists of 41 concise and clear items, and the total time required to complete the survey is 10 min or less (Adams et al., 2006; Mistades et al., 2011; Appendix A). This study was done for both countries, i.e., Malaysia and Korea, because even though both countries implemented STEM and STEAM for more than 10 years, many teachers and students are struggling with curriculum achievement and the progress is considered slow (Shahali et al., 2017; Kang, 2019).

**Figure 1:**
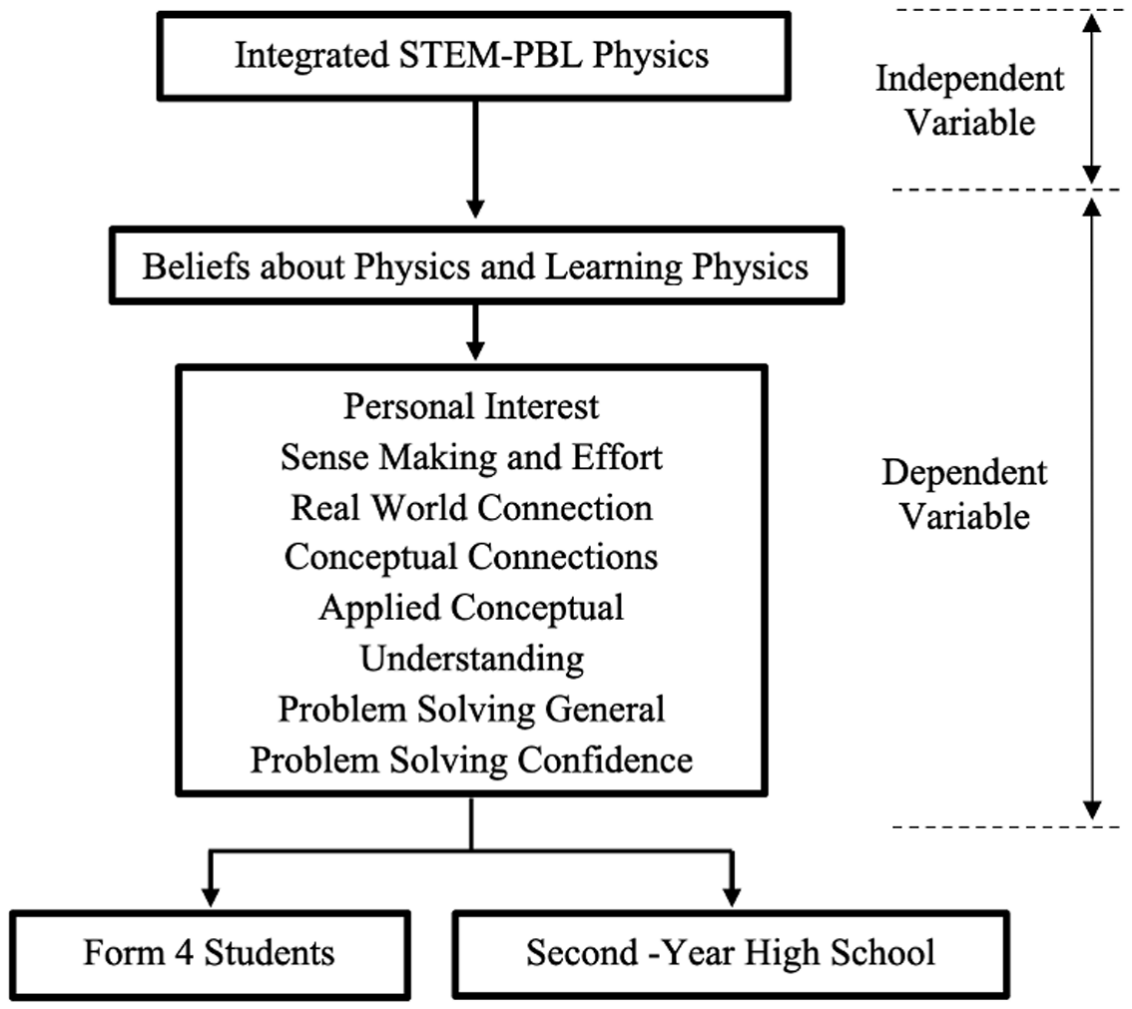
Research conceptual framework.

CLASS, initially in English, was translated into both Malay and Korean through a rigorous translation process called forward translation and back translation by two language experts in each research area to maintain the originality of CLASS (Bowles and Stansfield, 2008). The quantitative data were analyzed through SPSS Version 26.0. Figure 1 shows the conceptual framework used in this research. The independent variable is the integrated STEM-PjBL Physics Module. In contrast, the dependent variables are the eight subcategories of beliefs about physics and learning physics, e.g., personal interest, sense-making and effort, real-world connection, applied conceptual understanding, problem-solving general, problem-solving confidence, and problem-solving sophistication.

## 4. Results and analysis – Inferential statistical analysis

A paired samples *t*-test was conducted to evaluate the impact of integrated STEM-PjBL physics module intervention on students’ beliefs about physics and learning physics based on the students’ scores in CLASS and the results of the test are shown in Table 3. In terms of Malaysian students’ perspective, there was a statistically difference increase in beliefs about physics and learning physics in the experimental group from the pre-survey (*M* = 3.23, SD = 0.17) to the post-survey (*M* = 4.11, SD = 0.15), *t* (43) = −23.89, *p* < 0.001 (two-tailed). The mean increase was 0.88 with a 95% confidence interval ranging from −0.96 to −0.81. In addition, there was no statistically difference decrease in beliefs about physics and learning physics in the control group from the pre-survey (*M* = 3.25, SD = 0.19) to the post-survey (*M* = 3.23, SD = 0.17), *t* (43) = 0.31, *p* = 0.760 (two-tailed). The mean decrease was 0.02 with a 95% confidence interval ranging from −0.06 to 0.08.

**Table 3:** Results of paired samples *t*-test for students’ beliefs about physics and learning physics.

Group		Survey	Mean	SD	*t*	DF	*p* (2-tailed)	Mean difference
Malaysian student	CG (*N* = 44)	Pre-survey	3.25	0.19	0.31	43	0.760	0.02
		Post-survey	3.23	0.17				
	EG (*N* = 44)	Pre-survey	3.23	0.17	−23.89	43	<0.000*	−0.88
		Post-survey	4.11	0.15				
Korean student (*N* = 33)	CG (*N* = 33)	Pre-survey	3.10	0.17	0.82	32	0.420	0.03
		Post-survey	3.07	0.16				
	EG (*N* = 33)	Pre-survey	3.05	0.16	−15.45	32	<0.000*	−0.36
		Post-survey	3.41	0.17				

*Shows significant difference at *p* < 0.001.

EG, Experimental Group; CG, Control Group; Malaysian Student, Form Four; Korean Student, Second Year.

In terms of Korean students’ perspective, there was a statistically difference increase in beliefs about physics and learning physics in the experimental group from the pre-survey (*M* = 3.05, SD = 0.16) to the post-survey (*M* = 3.41, SD = 0.17), *t* (32) = −15.45, p < 0.001 (two-tailed). The mean increase was 0.36 with a 95% confidence interval ranging from −0.41 to −0.31. In addition, there was no statistically difference decrease in beliefs about physics and learning physics in the control group from the pre-survey (*M* = 3.10, SD = 0.17) to the post-survey (*M* = 3.07, SD = 0.16), *t* (32) = 0.82, *p* = 0.420 (two-tailed). The mean decrease was 0.03 with a 95% confidence interval ranging from −0.04 to 0.09.

*H1*: There is no significant difference in beliefs about physics and learning physics between pre-survey and post-survey for control group among Malaysian students and Korean students.

*H2*: There is no significant difference in beliefs about physics and learning physics between pretest and posttest for experimental group among Malaysian students and Korean students.

H1 is *accepted* - There is no significant difference in beliefs about physics and learning physics between pre-survey and post-survey for control group among Malaysian students and Korean students.

H2 is *rejected* - There is significant difference in beliefs about physics and learning physics between pre-survey and post-survey for experimental group among Malaysian students and Korean students.

An independent samples *t*-test was also conducted to compare students’ beliefs about physics and learning physics between the experimental group and the control group after the intervention (post-survey) based on the students’ scores in CLASS and the results of the survey are shown in Table 4. In terms of Malaysian students’ perspective, there was a statistically significant difference in beliefs about physics and learning physics between the experimental group (*M* = 4.11, SD = 0.15) and the control group (*M* = 3.23, SD = 0.17) in the post-survey, *t* (86) = 25.12, *p* < 0.001 (two-tailed). In addition, the assumption of homogeneity of variances was tested and not violated *via* Levene’s Test, *F* (86) = 0.88, *p* = 0.351. The magnitude of the difference in the means (mean difference = 0.88, 95% CI: 0.80 to 0.94) indicated a large effect size with Cohen’s d = 5.42.

**Table 4:** Results of independent samples *t*-test for students’ beliefs about physics and learning physics.

Group		M	SD	Levene’s test	*t*-test				
				*F*	*p*	*t*	DF	*P* (2-tailed)	Mean difference
Malaysian student	CG (*N* = 44)	3.23	0.17	0.88	0.351	25.12	86	<0.000*	0.88
	EG (*N* = 44)	4.11	0.15						
Korean student	CG (*N* = 33)	3.07	0.16	0.28	0.599	8.24	64	<0.000*	0.34
	EG (*N* = 33)	3.41	0.17						

*Shows significant different at *p* < 0.001.

EG, Experimental Group; CG, Control Group; Malaysian Student, Form Four; Korean Student, Second year.

In terms of Korean students’ perspective, there was a statistically significant difference in beliefs about physics and learning physics between the experimental group (*M* = 3.41, SD = 0.17) and the control group (*M* = 3.07, SD = 0.16) in the post-survey, *t* (64) = 8.24, *p* < 0.001 (two-tailed). In addition, the assumption of homogeneity of variances was tested and not violated *via* Levene’s Test, *F* (64) = 0.28, *p* = 0.599. The magnitude of the difference in the means (mean difference = 0.34, 95% CI: 0.26 to 0.42) indicated a large effect size with Cohen’s d = 2.06.

The results of the inferential statistical on the quantitative data showed that integrated STEM-PjBL physics module was able to give a significant improvement toward Form 4 and the second-year high school students’ beliefs about physics and learning physics. Meanwhile, traditional instruction showed no influence on students’ beliefs about physics and learning physics.

*H3*: There is no significant difference in beliefs about physics and learning physics between the experimental group and the control group after the post-survey among Malaysian students and Korean students.

H3 is *rejected* - There is significant difference in beliefs about physics and learning physics between the experimental group and the control group after the posttest among Malaysian students and Korean students.

### 4.1. Analysis of hypothesis

It is not surprising that H3 is rejected. There are many benefits STEM-PjBL offer to students in learning (Uden and Beaumont, 2006). These include:

- STEM-PjBL embodies the principles of constructivist learning
- STEM-PjBL promotes critical thinking skills in students
- STEM-PjBL promotes team working skills
- STEM-PjBL promotes deep learning
- STEM-PjBL helps students to develop metacognitive skill
- STEM-PjBL promotes problem solving skills

From the neuroscience perspectives, the following reasons are why STEM -PjBL was considered to be a better approach for students to learn physics.

i. Collaborative Learning Reduces Stress

Emotion plays a crucial role in learning. According to Kaufer (2011), the idea that how we feel influences how we are able to learn known as the “affective filter hypothesis,” stress, our emotion state influences learning, memory and decision making. In neuroscience, stress activates the amygdala, the segment of the brain connected with emotions and fear. The amygdala sends information to the hippocampus, the brain region associated with learning and memory. We learn and remember differently when the amygdala is firing. Kaufer (2011) argues that the stress response - popularly known as the “fight or flight” response — is chemically understood as the production of a variety of hormones, most significantly cortisol. When the stress is related to an emergency, cortisol is released by the adrenal gland into the brain to help us to combat or avoid the situation. But in chronic stress, the amygdala is constantly activated that has a negative effect on decision making resulting in decreased ability in learning.

In STEM-PjBL, as the students are working together and sharing knowledge, the burden of decision making is no longer falls on a single individual. It is a shared decision and thus reducing the stress that otherwise would happen.

ii. STEM-PjBL is Active Learning

Voss et al. (2011) argue that there is a difference between passive and active learning from a neurobiological perspective. They argued that volitional control is an omnipresent determinant of exploratory behaviors that occur whenever an organism is unconstrained in interactions with the environment. According to Kaufer (2011), optimized learning is produced in active learning when there is recruitment of multiple cortical areas and cross talk with the hippocampus in the brain. Kaufer (2011) furthers argues Active learning (volitional control) is advantageous for learning because distinct neural systems related to executive functions (planning or predicting, attention and object processing) are dynamically activated and communicate with the hippocampus, to enhance its performance.

iii. *STEM-PjBL Enables Students to Generate Information*

In STEM PjBL, students can generate information by linking new information to knowledge they already have because this activates our hippocampus. This happens through social information where students link their knowledge with knowledge that other students share as well as knowledge builds on knowledge known as metacognition (Voss et al., 2011).

iv. Learning in STEM PjBL is About Solving Problems

Traditional learning is where someone is told what someone else wants them to know and then the former is expected to transfer that knowledge into the workplace. Neuroscience shows that people are far more motivated to change their behavior and to adopt new ways of working when they have the insight from themselves. Creating insight requires a very different approach to delivering information. The information needs to be put in context for the learner. The learner then needs help to experience for themselves their new understanding followed by helping them to think about how they can apply their new understanding to their own role or their job.

Neuroscience indicates that a different way of designing and delivering learning is required. The emphasis now needs to be on how to get people’s attention and how they can retain what they have learned. Engagement is essential to applying what has been learned. If people understand what their learning means in practical terms to their job, have clear goals about what to do with their learning and get a sense of reward for adopting new behaviors, then what they have learned is far more likely to stick.

v. Neuro-Scientific Principles Complement and Connect with Socio-Constructivist Principles of Project-Based Learning

The well-established socio-constructivist principles of PjBL are closely connected and complementary with neuro-scientific principles of teaching and learning. It postulates that student constructs knowledge based on the prior knowledge and experiences of the learners. In STEM-PjBL, Learners also exchange experiences with their peers (Savery and Duffy, 1995; Richardson, 2003).

### 4.2. STEM PjBL guidelines for learning from neuroscience

Principles of neuroscience can be used by teachers to help students to learn better. Firstly, understanding how the brain works helps the teacher to plan lessons and choose methods that align with neuroscience research for learning. Secondly, research from neuroscience can help teachers to understand how the behavior of students is influenced by how the brain works and environment, genetics, and perceptions. Thirdly, research from neuroscience enables us to shed light on important topics related to how the brain learns such as including neuroplasticity, memory, metacognition, mindfulness, retrieval strategies, reflection, motivation, and prior knowledge. Fourthly, neuroscience helps us to understand how students’ brains are affected by factors such as emotion, exercise, sleep, motivation, and social encounters, to help us to choose the best help to give to students (Uden et al., 2022). The following principles from neuroscience can be used to help students to implement STEM-PjBL.

- Prior knowledge is important

Neuroscience studies (Bransford et al., 2000) revealed that the learning process leads to the creation of connections between several neural networks of different brain areas (Morris et al., 1988). Neurons connect each other by means of gates that are functionally modulated by neurotransmitters in the so-called synaptic junctions (Beale and Jackson, 1990). The long-lasting learning occurs when the connections between the neurons are strong and the networks are wide (Sousa, 2010; Fregni, 2019). It is important to link learning with prior knowledge.

- Use images to help students to understand abstract concepts.

The reason is that neuroscience research reveals that images such as comics help students to understand abstract concepts by making connections with real world situations (Bolton-Gary, 2012).

- Rehearsal information regularly

Because the synaptic strengthening between neurons may be weaken over time. It is important to retrieve information periodically (Karpicke et al., 2009). There must be opportunities for given to students by teachers to retrieve the concepts taught so as to allow metacognition to strengthen the connections between the neural networks. Teachers should change the type and duration of stimulus regularly

- Attention is important in learning.

According to neuroscience research, (Sousa, 2010), the teacher should change the type and the duration of the stimulus to foster learning because our brain filters out constant and repetitive information (Fregni, 2019).

- Pay attention to stress and anxiety

Research from neuroscience consider stress and anxiety are important factors that can affect learning. According to Fregni (2019), Too little and too much stress decrease learning. Moderate stress is beneficial if related to the learning context.

- The neuroscience of motivation

According to Willis (2010), intrinsic motivation is promoted by dopamine, a brain chemical that gives us a rush of satisfaction upon achieving a goal we have chosen. When dopamine levels rise, so does one’s sense of satisfaction and desire to continue to sustain attention and effort. Increased dopamine can also improve other mental processes, including memory, attention, perseverance, and creative problem-solving.

Willis (2019) argues that meeting desired choices, interacting with peers, movement, etc. releases Dopamine in the brain. It is possible to help students to maintain or boost motivation by knowing what boosts students’ dopamine levels. Giving choice to students can be used to increase students’ level of intrinsic motivation. This helps to shift responsibility for learning to students who now own the learning. Students will learn to develop the skills of evaluating, selecting, and following through with good choices (Willis, 2019)

- *Neuroscience principles for engagement and retention* The following principles from neuroscience can be used by teachers to promote engagement and retention in students (Ovation, 2021).

i. Break content into bite-sized chunks

Chunking can be used to help students to remember. Chunking is needed because the number of information a person can hold is seven, plus or minus two. Chunking allows the brain to digest and assimilate content better by making it easier to integrate to our long-term memory.

ii. Introduce a jolt

Human attention span is only 10 to 15 min. Attention is greater when we can introduce something new or different such as visual aid or humor, thus breaking the boredom.

iii. Enhance the relevancy of learning

It is important to show the learners what is relevant and important at the first 5 min of the lesson. This is because relevance plays a crucial role in cognition. When information is perceived as relevant, cognitive efforts significantly increase, leading to much higher cognitive effects.

iv. The Spacing effect

Learning should be spaced out. Crammed, intense learning over an extended period causes the brain to take in fewer facts. Students learn better by spreading out the lesson and review over time instead of engaging in one-time, overloaded top-down sessions.

v. Create a multisensory experience Students learn best when all their senses are engaged rather than using one sense.
vi. Trigger the right emotions

Emotion affects learning. It is important to encourage learners and make sure they feel welcome and cared for. Triggering the right emotions can help attendees learn better and increase overall engagement during a session.

## 5. Discussion

This study demonstrates the effect of integrated STEM-PjBL physics learning to students’ beliefs about physics and learning physics. Our Findings show that integrated STEM-PjBL physics learning intervention resulted in a positive shift in students’ beliefs about physics and learning physics, but the traditional instruction shows no influence on students’ beliefs about physics and learning physics for both Malaysian and Korean perspectives. Physics instruction is the significant factor that affects the shift in students’ beliefs about physics and learning physics (Hammer, 1994; Wieman and Perkins, 2005; Madsen et al., 2015).

There has been much research carried out on STEM-PjBL that show positively shifted student beliefs in various ways, For example, Han’s (2017) study showed that students who were positive toward PjBL components (i.e., technology-based learning, self-regulated learning, and hands-on activities) were more likely to have the intent to pursue a STEM-PjBL. STEM-project-based learning increases effectiveness, creates meaningful learning and influences student attitudes in future career pursuit; (Samsudin et al., 2017). Diana et al. (2021) findings show the effectiveness of the application of PjBL in STEM learning that improve students’ cognitive, affective, and psychomotor abilities; whereas in Bhakti et al. (2020) study, they found that STEM-PjBL, improved student science process skills in all indicators of the science process skills, where students also give a positive response to learning, because they feel they have more understanding, improved motivation and learning interests.

Our study is unique in that we want to investigate if there was any shift between traditional teaching and the use of STEM-PjBL by students in their belief in physics and learning physics. Our result clearly reveals that our STEM-PjBL shows a significant positive shift in students’ belief in physics and learning physics after being exposed to the STEM-PjBL approach. Another important difference between our study and others is that we have incorporated neuroscience research in our implementation of STEM-PjBL. Educational neuroscience, the study of the brain’s development, structure, and function, is a powerful discipline that can be very helpful to teachers to help students to learn better.

The positive shift of students; belief in physics and learning physics can be explained by the principles of educational neuroscience. Students at the STEM-PjBL class learned well because the learning was active. According to neuroscience active learning experiences promote changes in neural connections that are fundamental for learning in the brain. Simply listening to a lecture will not lead to learning. Neuroscience research shows us that active engagement such as facilitation in PjBL is a powerful way to learning.

In STEM-PjBL, the recall of prior knowledge is important, students were constantly challenged about what they knew. Students should be stimulated to connect the new concepts with the concepts they already knew (Sousa, 2010). By doing it, the students create new neural network paths and create a more distributed network that facilitates long lasting learning (Draganski et al., 2004). The synaptic strength in our brain may be weaken over time. To overcome this, it is necessary to retrieve the information periodically. It is important that we provide opportunities for retrieving the concepts learned to allow metacognition to strengthen the connections between the neural networks. In STEM-PjBL, this was happening all the time when students challenged each other to solve the problem as well as with the teacher.

Additionally, In STEM-PjBL students took control of their own learning, and they were able to make choices to engage in learning and received immediate feedback on their progress toward their chosen goals. This motivated them. When students interacted with their peers, working on challenging problems, their dopamine levels increased, and this help them to maintain their motivation. The brain is the core of human thought, consciousness, emotion, and memory. It is only reasonable that we apply the principles of neurosciences to help our students to learn better. Our research has found that by incorporating principles of neuroscience have impacted student shift in physics and learning physics.

## 6. Conclusion

Our study shows that integrated STEM-PjBL physics learning has significantly improved Form 4 and second-year high school students’ beliefs about physics and learning physics after the intervention. Students bring their existing beliefs about physics and learning to the classroom in which these beliefs may affect learning and how they interpret what they have learned in a physics class. This study applied integrated STEM education based on the principles of neuroscience in the form of interdisciplinary approach through PjBL to learn classical mechanics in secondary education in Malaysia and Korea. We did this this because integrated STEM education at the secondary education level is not well established in Malaysia although the Ministry of Education Malaysia has introduced the Malaysia Education Blueprint (2013–2025) to promote STEM education among secondary school students since 2013. At the same time, the Ministry of Education Korea has also issued a nationwide policy since 2011 to promote integrated STEAM education in secondary education that focuses on multidisciplinary approach. Despite the increase in STEAM education efforts, numerous studies have reported Korean teachers’ difficulties with integrated STEAM education especially in implementing a multidisciplinary approach. In recent years, the interdisciplinary approach is getting more attention in Korea, but limited research on the effect of the interdisciplinary nature of STEAM. It is important to investigate if integrated STEM-PjBL physics learning by students in both Malaysia and Korea would improve their belief about physics and physics learning based on the principles from neuroscience. Our study gives us positive outcome in both countries. Moreover, in our study we have identified principles from neuroscience that have important implications to help teachers to implement STEM-PjBL in physics learning.

Although the sample is small, we believe that our approach can be used by teachers who want to teach physics to students. This approach will help students to improve their belief about physics and learning physics. More empirical studies are needed to validate the approach. We are currently expanding the framework to the teaching of other subjects such as Chemistry and Mathematics. Further studies will be to incorporate Technology Pedagogy Content Knowledge (TPACK) to our framework for on demand online learning to meet the current trends of online learning due to the pandemic.

## Data availability statement

The original contributions presented in the study are included in the article/Supplementary material, further inquiries can be directed to the corresponding author.

## Ethics statement

The studies involving human participants were reviewed and approved by Educational Planning and Research Division, Ministry of Education (MoE), Malaysia. Written informed consent to participate in this study was provided by the participants’ legal guardian/next of kin.

## Author contributions

LU and FS: conceptualization. FS and JR: methodology, validation, formal analysis, investigation, resources, and data curation. JR: software and visualization. LU, FS, and GC: writing, supervision, and writing–editing. LU and GC: literature review. FS: project administration and funding acquisition. All authors contributed to the article and approved the submitted version.

## Funding

This work was supported by the Research Management Center Universiti Malaysia Sabah, Grant Number: UMSGreat0210-1/2018.

## Conflict of interest

The authors declare that the research was conducted in the absence of any commercial or financial relationships that could be construed as a potential conflict of interest.

## Publisher’s note

All claims expressed in this article are solely those of the authors and do not necessarily represent those of their affiliated organizations, or those of the publisher, the editors and the reviewers. Any product that may be evaluated in this article, or claim that may be made by its manufacturer, is not guaranteed or endorsed by the publisher.

## References

[ref1] Adams W. K. Perkins K. K. Podolefsky N. S. Dubson M. Finkelstein N. D. Wieman C. E. (2006). New instrument for measuring student beliefs about physics and learning physics: the Colorado learning attitudes about science survey. Phys. Rev. Phys. Educ. Res. 2, 1–14. doi: 10.1103/PhysRevSTPER.2.010101

[ref2] Angell C. Guttersrud Ø. Henriksen E. K. Isnes A. (2004). Physics: frightful, but fun - pupils’ and teachers’ views of physics and physics teaching. Sci. Educ. 88, 683–706. doi: 10.1002/sce.10141

[ref3] Atan H. Sulaiman F. Idrus R. M. (2005). The effectiveness of problem-based learning in the web-based environment for the delivery of an undergraduate physics course. Int. Educ. J. 6, 430–437. Available at: https://eric.ed.gov/?id=EJ854996

[ref4] Aviyanti L. (2020). *An Investigation into Indonesian Pre-service Physics Teachers' Scientific Thinking and Conceptual Understanding of Physics, Doctoral Dissertation*. Flinders University, Flinders Learning Exchange. Available at: https://flex.flinders.edu.au/file/a44a1398-06d4-451f-808c-6e1a702e060b/1/AviyantiThesis2020_LibraryCopy.pdf

[ref5] Av-Ron E. Byrne J. H. Baxter D. A. (2006). Teaching basic principles of neuroscience with computer simulations. J. Undergrad. Neurosci. Educ. 4, A40–A52. 23493644 PMC3592631

[ref6] Bakar N. I. Noordin N. Razali A. B. (2019). Effectiveness of project-based learning in improving listening competency among ESL learners at a Malaysian TVET college. Engl. Teach. 48, 11–28.

[ref013] Baran M. Maskan A. Yasar S. (2018). Learning physics through project-based learning game techniques. Int. J. Instr. 11, 221–234.

[ref7] Barnes P. (2019). *Make Educational Neuroscience Work in Your School–7 Tips*. Available at: https://blog.learnfasthq.com/make-educational-neuroscience-work-in-your-school-7-tips

[ref8] Beale R. Jackson T. (1990). Neural Computing-an Introduction. New York, USA: CRC Press.

[ref9] Bear M. F. Connors B. W. Paradiso M. A. (2015). Neuroscience: Exploring the Brain. Burlington, Massachusetts: Jones & Bartlett Learning.

[ref10] Bhakti Y. B. Astuti I. A. D. Okyranida I. Y. Asih D. A. S. Marhento G. Leonard L.. (2020). Integrated STEM project based learning implementation to ImproveStudent science process skills. February 2020. J. Phys. Conf. Ser. 1464:012016. doi: 10.1088/1742-6596/1464/1/012016

[ref11] Bocaneala F. (2015). Individual and Group Learning in Physics Education. [Doctoral Thesis]. The Ohio State University. Available at: https://etd.ohiolink.edu/apexprod/rws_etd/send_file/send?accession=osu1117151049&disposition=inline

[ref12] Bolton-Gary C. (2012). Connecting through comics: expanding opportunities for teaching and learning. US China Educ. Rev. 4, 389–395.

[ref13] Bowers J. S. (2016). The practical and principled problems with educational neuroscience. Psychol. Rev. 123, 600–612. doi: 10.1037/rev0000025, PMID: 26938449

[ref14] Bowles M. Stansfield C. (2008). A Practical Guide to Standards-Based Assessment in the Native Language. University of Illinois at Urbana-Champaign, Urbana, IL.

[ref15] Bransford J. D. Brown A. L. Cocking R. R. (2000). How People Learn. Washington, USA: National Academy Press.

[ref16] Brown D. E. (2014). Using Analogies and Examples to Help Students Overcome Misconceptions in Physics: A Comparison of Two Teaching Strategies. [Doctoral Dissertation 1896–February, No. 4249]. Available at: https://scholarworks.umass.edu/dissertations_1/4249

[ref17] Brown R. E. (2019). Why study the history of neuroscience? Front. Behav. Neurosci. 13:82. doi: 10.3389/fnbeh.2019.00082, PMID: 31191266 PMC6539194

[ref18] Brown-Martin G. (2018). Education and the fourth industrial revolution (learning to thrive in a transforming world). In *Proceeding the 11th Annual International Conference of Education, Research and Innovation (ICERI) 2018*, Seville, Spain, 12–14 November 2018.

[ref19] Bruer J. T. (1997). Education and the brain: a bridge too far. Educ. Res. 26, 4–16. doi: 10.3102/0013189X026008004

[ref20] Bunyamin M. A. H. Finley F. (2016). STEM Education in Malaysia: Reviewing the Current Physics Curriculum. *Paper Presented at The International Conference of Association for Science Teacher Education*, Reno, Nevada.

[ref21] Bunyamin M. A. H. Talib C. A. Ahmad N. J. Ibrahim N. H. Surif J. (2020). Current teaching practice of physics teachers and implications for integrated STEM education. Univ. J. Educ. Res. 8, 18–28. doi: 10.13189/ujer.2020.081903

[ref07] Chang W. (2005). Impact of constructivist teaching on students’ beliefs about teaching and learning in introductory physics. J. Sci. Math. Technol. Educ. 5, 95–109.

[ref22] Chala A. A. Kedir I. Wami S. (2020). Secondary School Students’ Beliefs towards Learning Physics and its Influencing Factors Research on Humanities and Social Sciences. Res. Human. Soci Science. 10, 37–49.

[ref23] Clement N. D. Lovat T. (2012). Neuroscience and education: issues and challenges for curriculum. Curric. Inq. 42, 534–557. doi: 10.1111/j.1467-873X.2012.00602.x

[ref24] Diana N. Yohannes Y. Sukma Y. (2021). The effectiveness of implementing project-based learning (PjBL) model in STEM education: a literature review. J. Phys. Conf. Ser. 1882:012146. doi: 10.1088/1742-6596/1882/1/012146

[ref25] Donley R. D. Ashcraft M. H. (1992). The methodology of testing naive beliefs in the physics classroom. Mem. Cogn. 20, 381–391. doi: 10.3758/BF03210922, PMID: 1495400

[ref26] Draganski B. Gaser C. Busch V. Schuierer G. Bogdahn U. May A. (2004). Changes in grey matter induced by training. Nature 427, 311–312. doi: 10.1038/427311a, PMID: 14737157

[ref27] Fatin A. P. M. Salleh A. M. Bilal A. Salmiza S. (2012). *Faktor Penyumbang Kepada Kemerosotan Penyertaan Pelajar Dalam Aliran Sains: Satu Analisis Sorotan Tesis*. MEDC.

[ref28] Feiler J. Stabio M. (2018). Three pillars of educational neuroscience from three decades of literature. Educ. Neurosci. Rev. 13, 17–25. doi: 10.1016/j.tine.2018.11.001

[ref29] Fischer K. W. Goswami U. Geake J. (2010). The future of educational neuroscience. Mind Brain Educ. 4, 68–80. doi: 10.1111/j.1751-228X.2010.01086.x, PMID: 36921791

[ref30] Fregni F. (2019). Critical Thinking in Teaching and Learning: The Nonintuitive New Science of Effective Learning. Boston, USA: Lumini LLC.

[ref31] Gabrieli J. D. (2016). The promise of educational neuroscience: Comment on Bowers (2016). Psychol. Rev. 123, 613–619. doi: 10.1037/rev0000034 27657440

[ref32] Goswami U. (2004). Neuroscience and education. Br. J. Educ. Psychol. 74, 1–14. doi: 10.1348/000709904322848798, PMID: 15096296

[ref33] Goswami U. (2006). Neuroscience and education: from research to practice? Nat. Rev. Neurosci. 7, 406–413. doi: 10.1038/nrn1907, PMID: 16607400

[ref34] Goswami U. (2020). *What is Neuroscience?* Available at: https://www.thebritishacademy.ac.uk/blog/what-is-neuroscience/ (Accessed November 8, 2022).

[ref35] Hairan A. M. Abdullah N. Abdullah A. H. (2018). Conceptual understanding of Newtonian mechanics among afghan students. European. J. Phys. Educ. 10, 1–12.

[ref110] Halim L. Rahman N. A. Ramli N. A. M. Mohtar L. E. (2018). “Influence of students’ STEM self-efficacy on STEM and physics careerchoice,” in AIP Conference Proceedings 1923:020001. AIP Publishing. doi: 10.1063/1.5019490

[ref016] Halloun I. Hestenes D. (1996). Views about Sciences Survey: VASS. Paper presented at the Annual Meeting of the National Association for Research in Science Teaching. Saint Louis, United States of America. Available at: https://eric.ed.gov/?id=ED394840

[ref36] Hammer D. (1994). Epistemological beliefs in introductory physics. Cogn. Instr. 12, 151–183. doi: 10.1207/s1532690xci1202_4, PMID: 32297037

[ref37] Han S. (2017). Korean students’ attitudes toward STEM project-based learning and major selection. Educ. Sci. Theory Pract. 17, 529–548. doi: 10.12738/estp.2017.2.0264

[ref38] Harris A. D. Bradham D. D. Baumgarten M. Zuckerman I. H. Fink J. C. Perencevich E. N. (2004). The use and interpretation of quasi-experimental studies in infectious diseases. Antimicrob. Resist. 38, 1586–1591. doi: 10.1086/420936 15156447

[ref39] Hirschfeld D. (2012). *Interest in Science Careers Wanes in Latin America*. Available at: http://www.scidev.net/global/capacity-building/news/interest-in-science-careers-wanes-in-latin-america.html# (Accessed November 10, 2016).

[ref40] Holubova R. (2008). Effective teaching methods: project-based learning in physics. US China Educ. Rev. 5, 27–36.

[ref41] Howard-Jones P. A. Varma S. Ansari D. Butterworth B. De Smedt B. Goswami U.. (2016). The principles and practices of educational neuroscience: comment on bowers. Psychol. Rev. 123, 620–627. doi: 10.1037/rev0000036 27657441

[ref42] Howard-Jones P. Pickering S. Diack A. (2007). *Perception of the Role of Neuroscience in Education*. Summary Report for the DfES Innovation Unit.

[ref43] Jho H. Hong O. Song J. (2016). An analysis of STEM/STEAM teacher education in Korea with a case study of two schools from a community of practice perspective. Eur. J. Math. Sci. Technol. Educ. 12, 1843–1862. doi: 10.12973/eurasia.2016.1538a

[ref44] Josiah M. M. (2013). Effects of practical physics knowledge on students’ academic achievement: a study of Pankshin local government area of plateau state, Nigeria. World Educ. Forum 2, 1–9.

[ref45] Kandel E. R. Koester J. D. Mack S. H. Siegelbaum S. A. (2021). Principles of Neural Science. 6th. New York: McGraw Hill.

[ref46] Kang N. H. (2019). A review of the effect of integrated STEM or STEAM (science, technology, engineering, arts and mathematics) education in South Korea. Asia Pac. Sci. Educ. 5, 1–22. doi: 10.1186/s41029-019-0034-y

[ref47] Karam R. A. S. Lima N. W. (2022). *Using History of Physics to Teach Physics?* In: Connecting Research in Physics Education with Teacher Education 3. International Union of Pure and Applied Physics (IUPAP).

[ref48] Karamustafaoglu O. (2009). Active learning strategies in physics teaching. Energy Educ. Sci. Technol. B Soc. Educ. Stud. 1, 27–50.

[ref49] Karpicke J. D. Butler A. C. Roediger H. L. (2009). Metacognitive strategies in student learning: do students practise retrieval when they study on their own? Memory 17, 471–479. doi: 10.1080/09658210802647009, PMID: 19358016

[ref50] Kaufer D. (2011). Daniela Kaufer: What can Neuroscience Research Teach Us about Teaching?. Available at: https://gsi.berkeley.edu/programs-services/hsl-project/hsl-speakers/kaufer/

[ref09] Kiong S. S. Sulaiman S. (2010). “Study of epistemological beliefs, attitudes towards learning and conceptual understanding of newtonian force concept among physics education undergraduates” in Universiti Teknologi Malaysia Institutional Repository (Skudai: Universiti Teknologi Malaysia).

[ref51] Kovanen A. (2011). *Where are we after 30 Years of Physics Education Research? (Unpublished Master’s Thesis). Centre for Teaching Excellence*. United States Military Academy, West Point, New York, USA.

[ref52] Larmer J. Mergendoller J. R. (2010). Seven essentials for project-based learning. Educ. Leadersh. 68, 34–37. Available at: https://www.ascd.org/el/articles/seven-essentials-for-project-based-learning

[ref53] Lee M. C. Fauziah S. (2018). The effectiveness of practical work on students’ motivation and understanding towards learning physics. Int. J. Hum. Soc. Sci. Invent. 7, 35–41. doi: 10.15242/dirpub.hdir1217224

[ref54] Lee B. Kim H. (2018). Trends of the research in physics education in Korea. J. Korean Phys. Soc. 72, 1502–1507. doi: 10.3938/jkps.72.1502, PMID: 36806667

[ref55] Madsen A. McKagan S. B. Sayre E. C. (2015). How physics instruction impacts students’ beliefs about learning physics: a meta-analysis of 24 studies. Phys. Rev. Phys. Educ. Res. 11:010115. doi: 10.1103/PhysRevSTPER.11.010115

[ref56] Mason L. (2009). Bridging neuroscience and education: a two-way path is possible. Cortex 45, 548–549. doi: 10.1016/j.cortex.2008.06.003, PMID: 18632093

[ref57] Meilleur C. (2022). *Branches of Neuroscience*. Knowledge One. Available at: https://knowledgeone.ca/4-branches-of-neuroscience/ (Accessed November 9, 2022).

[ref58] Meltzoff A. N. Kuhl P. K. Movellan J. Sejnowski T. J. (2009). Foundations for a new science of learning. Science 325, 284–288. doi: 10.1126/science.1175626, PMID: 19608908 PMC2776823

[ref01] Minsitry of Education, (2013–2025). Malaysia Education Blueprint 2013-2025 (Pre-School to Post Secodnary Education). Available at: https://www.moe.gov.my/menumedia/media-cetak/penerbitan/dasar/1207-malaysia-education-blueprint-2013-2025/file

[ref08] Mistades V. M. (2007). Exploring business students’ and liberal arts students’ beliefs about physics and physics learning. Asia Pac. Educ. Rev. 8, 100–106.

[ref018] Mistades V. Reyes R. D. Scheiter J. (2011). Transformative learning: shifts in students’ attitudes toward physics measured with the colorado learning attitudes about science survey. Int. J. Humanit. Soc. Sci. 1, 45–52.

[ref59] Morris R. G. Kandel E. R. Squire L. R. (1988). The neuroscience of learning and memory: cells, neural circuits and behavior. Trends Neurosci. 11:125. doi: 10.1016/0166-2236(88)90136-1, PMID: 36758544

[ref60] Musasia A. M. Abacha O. A. Biyoyo M. E. (2012). Effect of practical work in physics on girls’ performance, attitude change and skills Acquisition in the Form two-Form Three Secondary Schools’. Int. J. Humanit. Soc. Sci. 2, 151–166. Available at: http://www.ijhssnet.com/journals/Vol_2_No_23_December_2012/18.pdf

[ref61] Naudé W. (2017). *Entrepreneurship, Education and the Fourth Industrial Revolution in Africa (IZA Discussion Paper No. 10855)*.

[ref62] O’Neill D. Mcloughlin E. (2021). Examining students' interest in physics at second level in Ireland. J. Phys. Conf. Ser. 1929:012033. doi: 10.1088/1742-6596/1929/1/012033

[ref63] Olzan G. Bevins S. (Reviewing Editor). (2016). A project-based learning approach to teaching physics for pre-service elementary school teacher education students. Cogent Educ. 3:1200833, doi: 10.1080/2331186X.2016.1200833

[ref64] Ovation. (2021). *Neuroscience-Based Tips to Design Engaging Virtual Experiences*. Available at: https://ovationdmc.com/6-neuroscience-based-tips-to-design-engaging-virtual-experiences/

[ref65] Parmar P. (2022). 10 Effective Strategies to Use in Teaching Physics. Available at: https://classplusapp.com/growth/10-effective-strategies-to-use-in-teaching-physics/ (Accessed November 28, 2022).

[ref017] Perkins K. K. Gratny M. M. Adams W. K. Finkelstein N. D. Wieman C. E. (2006). Towards Characterizing the Relationship between Students’ Interest in and their Beliefs about Physics. AIP Conf. Proc. 818, 137–140. doi: 10.1063/1.2177042

[ref66] Petitto L. A. Dunbar K. (2004). “New findings from educational neuroscience on bilingual brains, scientific brains, and the educated mind” in Building Usable Knowledge in Mind, Brain, and Education. eds. Fischer K. Katzir T. (Cambridge: Cambridge University Press)

[ref67] Rabinovich M. I. Varona P. Selverston A. I. Abarbanel H. D. I. (2006). Dynamical principles in neuroscience. Rev. Mod. Phys. 78, 1213–1265. doi: 10.1103/RevModPhys.78.1213, PMID: 36921679

[ref015] Redish E. F. Saul J. M. Steinberg R. N. (1998). Student expectations in introductory physics. Am. J. Phys. 66, 212–224. doi: 10.1119/1.18847

[ref68] Reiner M. (1998). Thought experiments and collaborative learning in physics. Int. J. Sci. Educ. 20, 1043–1058. doi: 10.1080/0950069980200903, PMID: 35214484

[ref69] Richardson V. (2003). Constructivist pedagogy. Teach. Coll. Rec. 105, 1623–1640. doi: 10.1046/j.1467-9620.2003.00303.x, PMID: 36896973

[ref70] Riskawati N. Marisda D. H. (2022). “High school Students' interest in choosing physics as a major in college” in VCOSPILED 2021 2nd Virtual Conference on Social Science in Law, Political Issue and Economic Development (Makassar, Indonesia: Universitas Muhammadiyah Makassar)

[ref71] Romero S. (2019). *Neuroscience: Overview, History, Major Branches*. Mega Interesting. Available at: https://www.megainteresting.com/answers/questions-answers/neuroscience-overview-history-major-branches-781573144915 (Accessed November 10, 2022).

[ref06] Rose A. A. R. Mohamad S. R. Azlin N. M. Zarina O. Lyndon N. (2013). Inculcation of science process skills in a science classroom. Asian Soc. Sci. 9, 47–57. Available at: https://www.academia.edu/25251013/Inculcation_of_Science_Process_Skills_in_a_Science_Classroom

[ref72] Sahin M. (2010). Effects of problem-based learning on university students’ epistemological beliefs about physics and physics learning and conceptual understanding of Newtonian mechanics. J. Sci. Educ. Technol. 19, 266–275. doi: 10.1007/s10956-009-9198-7

[ref73] Samsudin M. A. Nurulazam M. A. Zain Jamali S. M. Ebrahim N. A. (2017). Physics achievement in STEM project-based learning (PjBL): a gender study. Asia Pac. J. Educ. Educ. 32, 21–28.

[ref012] Santyasa I. W. Rapi N. K. Sara I. W. W. (2020). Project based learning and academic procrastination of students in learning physics. Int. J. Instr. 13, 489–508. doi: 10.29333/iji.2020.13132a

[ref74] Savery J. R. Duffy T. M. (1995, 1995). Problem based learning: an instructional model and its constructivist framework. Educ. Technol. 35, 31–38.

[ref75] Shahali E. H. M. Ismail I. Halim L. (2017). STEM education in Malaysia: policy, trajectories and initiatives. Asian Policy Res. 8, 122–132. doi: 10.4324/9781003099888

[ref76] Sheldrake R. Mujtaba T. Reiss M. J. (2019). Students’ changing attitudes and aspirations towards physics during secondary school. Res. Sci. Educ. 49, 1809–1834. doi: 10.1007/s11165-017-9676-5

[ref77] Society for Neuroscience. (2022). *Neuroscience Core Concepts: The Essential Principles of Neuroscience*. Available at: https://www.brainfacts.org/-/media/Brainfacts2/Core-Concepts/NGSS-Core-Concepts.pdf (Accessed November 21, 2022).

[ref78] Sousa D. A. (2010). Mind, Brain, and Education: Neuroscience Implications for the Classroom. Bloomington, USA: Solution Tree Press.

[ref79] Squire L. R. Berg D. K. Bloom F. E. Du Lac S. Ghosh A. Spitzer N. C. (2013). Fundamental Neuroscience. Cambridge: Academic Press.

[ref80] Sumintono B. (2015). Science Education in Malaysia: Challenges in the 21^st^ Century. *The 1^st^ International Seminar on Science Education. Universitas Negeri Yogyakarta, Indonesia*. Available at: https://eprints.um.edu.my/15605/1/Science_education_in_Malaysia_Bambang_Sumintono_UM.pdf

[ref81] Suraya B. Norsalawati W. Nasir I. (2017). Integration of STEM education in Malaysia and why to STEAM. Int. J. Acad. Res. Bus. Soc. Sci. 7, 645–654. doi: 10.6007/IJARBSS/v7-i6/3027

[ref82] Sussman O. (2021). *Neuroscience: Overview, History, Major Branches. SimplyPsychology*. Available at: https://www.simplypsychology.org/neuroscience.html (Accessed November 8, 2022).

[ref83] The Scando Review. (2022). *Educational Neuroscience: Benefits, Challenges and Myths*. Available at: https://www.thescandoreview.com/p/educational-neuroscience#details

[ref84] Türk N. Kalaycı N. Yamak H. (2018). New trends in higher education in the globalizing world: STEM in teacher education. Univ. J. Educ. Res. 6, 1286–1304. doi: 10.13189/ujer.2018.060620

[ref85] Uden L. Beaumont C. (2006). “Why problem-based learning” in Technology and Problem-Based Learning (Pennsylvania, United States: IGI Global), 44–64.

[ref86] Uden L. Sulaiman F. Lamun R. F. (2022). Factors influencing students’ attitudes and readiness towards active online learning. Physics. Educ. Sci. 12:746. doi: 10.3390/educsci12110746

[ref87] Veronika A. T. Johannes V. D. W. Budijanto U. (2017). Application of direct instruction with laboratory activity to improve students’ participation and learning achievement. PEOPLE, 3, 1276–1284. doi:10.20319/pijss.2017.32.12761284

[ref88] Voss J. L. Gonsalves B. D. Federmeier K. D. Tranel D. Cohen N. J. (2011). Hippocampal brain-network coordination during volitional exploratory behavior enhances learning. Nat. Neurosci. 14, 115–120. doi: 10.1038/nn.2693, PMID: 21102449 PMC3057495

[ref89] Wang M. T. Chow A. Degol J. L. Eccles J. S. (2017). Does Everyone’s motivational beliefs about physical science decline in secondary school?: heterogeneity of adolescents’ achievement motivation trajectories in physics and chemistry. J. Youth Adolesc. 46, 1821–1838. doi: 10.1007/s10964-016-0620-1, PMID: 27909854 PMC5453850

[ref90] Webster D. B. (1999). Neuroscience of Communication. United States: Singular Publishing Group

[ref010] Wieman C. E. (2010). Teaching physics using PhET simulations. The Physics Teacher 48:225.

[ref91] Wieman C. E. Perkins K. K. (2005). Transforming physics education. Phys. Today 58, 36–42. doi: 10.1063/1.2155756, PMID: 36914806

[ref014] Widyaningsih S. W. Yusuf I. (2020). Implementation of project-based learning (pjbl) assisted by e-learning through lesson study activities to improve the quality of learning in physics learning planning courses. Int. J. High. Educ. 9, 60–68. doi: 10.5430/ijhe.v9n1p60

[ref92] Wilkinson J. W. (1999). The contextual approach to teaching physics Australian science teachers journal. Can. Underwrit. 45, 43–50.

[ref93] Williams C. Stanisstreet M. Spall K. Boyes E. Dickson D. (2003). Why aren’t secondary students interested in physics? Phys. Educ. 38, 324–329. doi: 10.1088/0031-9120/38/4/306

[ref94] Willis J. (2010). “The Current Impact of Neuroscience on Teaching and Learning” in Mind, Brain and Education: Neuroscience Implications for the Classroom. ed. D. A. Sousa ( Bloomington, IN: Solution Tree Press), 45–68.

[ref95] Willis J. (2019). *Maintaining Students’ Motivation for Learning as the Year Goes OnNeuroscience can Suggest Ways to Keep Students Working Toward their Learning Goals after their Initial Excitement Wears off*. Available at: https://www.edutopia.org/article/maintaining-students-motivation-learning-year-goes

[ref96] Ziad W. K. Md Norazam M. F. A. Kaco H. Mohd Idris F. Zulkefly N. R. Mohd S. M.. (2021). An evaluation of Student’s perception towards learning physics at lower secondary school. J. Pendidikan Sains Matematik Malaysia 11, 94–106. doi: 10.37134/jpsmm.vol11.sp.9.2021

